# Hepatic HMOX1 Expression Positively Correlates with Bach-1 and miR-122 in Patients with HCV Mono and HIV/HCV Coinfection

**DOI:** 10.1371/journal.pone.0095564

**Published:** 2014-04-21

**Authors:** Elżbieta Jabłonowska, Kamila Wójcik, Bożena Szymańska, Aleksandra Omulecka, Hanna Ćwiklińska, Anna Piekarska

**Affiliations:** 1 Department of Infectious Diseases and Hepatology, Medical University of Lodz, Łódź, Poland; 2 Central Laboratory, Medical University of Lodz, Łódź, Poland; 3 Department of Pathology, Biegański Provincial Specialistic Hospital, Łódź, Poland; 4 Laboratory of Neuroimmunology, Department of Neurology, Medical University of Łódź, Poland; Institut Pasteur, France

## Abstract

**Aim:**

To analyze the expression of HMOX1 and miR-122 in liver biopsy samples obtained from HCV mono-and HIV/HCV co-infected patients in relation to selected clinical parameters, histological examination and IL-28B polymorphism as well as to determine whether HMOX1 expression is dependent on Bach-1.

**Materials and Methods:**

The study group consisted of 90 patients with CHC: 69 with HCV mono and 21 with HIV/HCV co-infection. RT-PCR was used in the analysis of HMOX1, Bach-1 and miR-122 expression in liver biopsy samples and in the assessment of IL-28B single-nucleotide polymorphism C/T (rs12979860) in the blood. Moreover in liver biopsy samples an analysis of HO-1 and Bach-1 protein level by Western Blot was performed.

**Results:**

HCV mono-infected patients, with lower grading score (G<2) and higher HCV viral load (>600000 IU/mL) demonstrated higher expression of HMOX1. In patients with HIV/HCV co-infection, the expression of HMOX1 was lower in patients with lower lymphocyte CD4 count and higher HIV viral load. IL28B polymorphism did not affect the expression of either HMOX1 or miR-122. Higher HMOX1 expression correlated with higher expression of Bach-1 (Spearman’s ρ = 0.586, p = 0.000001) and miR-122 (Spearman’s ρ = 0.270, p = 0.014059).

**Conclusions:**

HMOX1 and miR-122 play an important role in the pathogenesis of CHC in HCV mono-and HIV/HCV co-infected patients. Reduced expression of HMOX1 in patients with HIV/HCV co-infection may indicate a worse prognosis in this group. Our results do not support the importance of Bach-1 in repression of HMOX1 in patients with chronic hepatitis C.

## Introduction

Hepatitis C virus infection (HCV) remains a worldwide health problem with about 3–4 million cases reported each year [1]. Approximately 80% of patients become chronically infected after acute infection, 20% of whom progress to cirrhosis [2]–[3]. The annual rate of hepatocellular carcinoma (HCC) in cirrhosis patients is about 3–8% [4].

Current therapy based on pegylated interferon, ribavirin and protease inhibitors like boceprevir or telaprevir is effective in no more than 70% of patients with CHC [5], [6].

Reactive oxygen species (ROS) production can suppress HCV replication [7]. However, excessive production may cause hepatocyte damage [8]–[11]. Hence, antioxidant enzymes, such as Heme oxygenase-1 (HO-1), induced in response to “stressful stimuli”, play a crucial role by suppressing inflammation and protecting against oxidative stress [12], [13].

However, the effect of HO-1 on HCV replication is unclear. While several experimental studies indicate that it plays an important role in the inhibition of HCV replication [14], [15], others indicate the contrary [16]. Moreover, experimental studies describing the increase of HCV replication under the influence of microRNA-122 (miR-122) are not always confirmed *in vitro* [17]. Interestingly, the antagomir of miR-122 has been found to increase HO-1 mRNA levels in CNS3 and NS3–5B Huh-7 cells [15].

The aim of this present study is to examine the expression of heme oxygenase-1 gene (HMOX1), BTB and CNC homology 1 (Bach-1) as well as miR-122 in liver biopsy specimens obtained from patients with CHC in relation to selected clinical parameters, histological examination and IL-28B polymorphism. In addition, Western-Blot analysis was used to investigate the levels of Bach-1 and HO-1 proteins in liver biopsy specimens.

## Materials and Methods

The study was approved by the Ethical Committee of the Medical University of Lodz. Informed written consent was obtained from all patients.

Liver biopsies were performed as part of routine standard of care for the subjects studied. The study group consisted of 90 patients: 69 with HCV mono-and 21 with HIV/HCV co-infection. The diagnosis of chronic hepatitis was based on the results of liver biopsy specimen examination. Patients with other systemic or inflammatory diseases, other causes of liver disease, previous immunosuppressive or anti HCV treatment and pregnant women were excluded from this study. Grade of inflammation and necrotic changes (grading-G) as well as stage of fibrosis (staging-S) were assessed according to the Batt and Ludwig scale [18]. RNA analyses were performed by use of 2-mm sections of liver that were excised from the core liver biopsy samples immediately after the biopsy procedure. These sections were immediately submerged in RNA later solution and stored at −70°C until use.

Plasma HCV viral load was measured within 3 months of liver biopsy and was determined by reverse transcription polymerase chain reaction (RT-PCR method; Cobas AmpliPrep/Cobas TaqMan HCV Test, Roche Diagnostics), HCV genotypes were determined by VERSANT HCV Genotype 2.0 Assay, LiPA test. Alanine aminotransferase (ALT) activity was measured 1–3 days before the liver biopsy, reference ranges for men and women were 0–41 U/L and 0–31 U/L, respectively. To diagnose HIV infection, an enzyme linked immunosorbent assay (ELISA test) and confirmatory W-Blot were used. In patients with HIV/HCV co-infection, HIV viral load (RT-PCR method, Cobas AmpliPrep/Cobas TaqMan HIV-1 Test, Roche Diagnostics) and lymphocytes CD4 count (using flow cytometry) were measured.

### Analysis of IL-28B Single-nucleotide Polymorphism C/T (rs12979860)

Genomic DNA was isolated from 200 µL of blood using the QIAamp DNA Blood Mini Kit (Qiagen) according to the manufacturer’s protocol. DNA was quantified using a PicoDrop spectrophotometer (Picodrop Limited). IL-28B single-nucleotide polymorphism C/T (rs12979860) was analyzed by means of Custom® SNP Genotyping Assays (Applied Biosystems). Primer and probe sequences were Forward Primer 5′GCCTGTCGTGTACTGAACCA, Reverse Primer 5′-GCGCGGAGTGCAATTCAAC, Probe (C allele) 5′-VIC-TGGTTCGCGCCTTC, Probe (T allele) 5′-FAM-CTGGTTCACGCCTTC. Genotyping was performed using the ABI7900HT Real-Time PCR System (Applied Biosystems) in 25 µL reaction volume containing 10 ng DNA, 12.5 µL TaqMan® Universal PCR Master Mix and 1.25 µL (40×) Custom® SNP Genotyping Assays and analyzed by means of Sequence Detection System 2.3 Software.

### Total RNA Isolation

Total RNA was extracted using the mirVana™ miRNA isolation kit (Ambion) according to the manufacturer’s instructions. Briefly, frozen samples were homogenized in 300 µl of Lysis/Binding Solution using a TissueRuptor homogenizer (Qiagen). RNA was eluted in 100 µl RNase-free water and quantified using PicoDrop spectrophotometer. The quality of RNA samples was analyzed by measuring the ratio of absorptions at 260/280 nm. The purified total RNA was immediately used for cDNA synthesis or stored at −80°C.

### miRNA-122 Expression

Reverse transcription was carried out on 10 ng of total RNA in 15 µl reactions using the TaqMan® MicroRNA Reverse Transcription Kit (Applied Biosystems) according to the manufacturer’s instructions. The RT reaction was diluted 10 times in nuclease-free water and 9 µL of aliquots were subsequently used for PCR amplification using TaqMan® MicroRNA Assays (miR-122 -Assay ID 002130, RNU 24 -Assay ID 001001), according to the manufacturer’s instructions (Applied Biosystems). RNU 24 (SNORD24 small nucleolar RNA, C/D box 24) was used as an endogenous control.

### mRNA Expression

Homo sapiens-specific TaqMan Gene Expression Assays (Applied Biosystems) for heme oxygenase (HMOX1, Hs01110250_m1), BTB and CNC homology 1 (Bach-1, Hs154276 _XX) were used. cDNA generation was performed using 250 ng of total RNA with High Capacity cDNA Reverse Transcription Kits according to the manufacturer’s protocols (Applied Biosystems). First-strand cDNA was subsequently diluted 10 times in nuclease-free water before addition to the RT-PCR reaction mixture. mRNA expression levels were analyzed using beta actin (ACTB) as an endogenous control.

### Real Time PCR Analysis

TaqMan PCR assays were performed in 96-well optical plates on 7900HT Fast Real-Time PCR System (Applied Biosystems) and were analyzed using Sequence Detection System 2.0 Software. Fold induction values were calculated according to the equation 2ΔΔCt, where ΔCt represents the differences in cycle threshold numbers between the target gene and endogenous control, and ΔΔCt represents the relative change in these differences between examined and control groups.

### Western Blot Analysis

W-Blot analysis was performed in 32 out of 90 patients. Samples were chosen based on the RT-PCR analysis performed previously. W-Blot was carried out in 10 patients with HCV monoinfection among whom 5 patients were found to have viremia higher than 600 000 IU/ml and HO-1 expression closest to the median for this group (the result obtained by RT-PCR). The remaining 5 patients had viremia below 600 000 IU/ml and HO-1 expression closest to the median for this group (the result obtained by RT-PCR). In the group with HIV/HCV coinfection W-Blot analysis was performed in 15 patients. In this group selection criteria for W-B analysis included CD4 count and HIV viremia.

Total homogenates of biopsy samples were resolved on SDS electrophoresis gels by standard procedures. Immunoblotting was performed with anti-HO-1 rabbit polyclonal antibody, ADISPA-896 (Enzo Life Sciences, Germany) or with anti-Bach1 goat polyclonal antibody, sc-14700 (Santa Cruz Biotechnology, INC). Final detection was achieved with the appropriate secondary antibody coupled to HRP (anti-rabbit IgG-HRP or anti-goat IgG-HRP, Santa Cruz Biotechnology, INC), followed by Western Lightning ECL Pro substrate (Perkin Elmer), and visualized by gel imaging system for chemiluminescence G:Box Chemi XR5 (Syngene).

Equal loading of proteins was checked by immunodetection of glyceraldehyde 3-phosphate dehydrogenase (GAPDH) mouse monoclonal Ab, MAB 374 (Millipore) and anti-mouse IgG-HRP as a secondary antibody (Santa Cruz Biotechnology, INC).

### Statistical Methods

Average values and standard deviation of quantitative traits were calculated for parameters with normal distribution. Variables that were not normally distributed were expressed as median, lower -upper quartiles (LQ-UQ). These variables were compared using the Mann-Whitney test for two groups and the Kruskal-Wallis test with post-hoc Conover-Inman test for more than two groups. The Chi-square distribution, Yates’ correction for continuity or Fisher’s exact test were used to compare categorical variables between groups, based on the size of the studied group. Spearman’s rank correlation coefficient was applied to determine the association between two quantities which were not normally distributed.

## Results

### Characteristics of the Study Group

The study group consisted of 90 patients: 69 with HCV mono-and 21 with HIV/HCV coinfection, of whom 27 were women and 63 were men. The median age of patients was 30 years (LQ 29 years-UQ34 years).

Among subjects with HIV/HCV co-infection 16 were receiving antiretroviral treatment, while 5 were not. The median CD4 cell count was 405 cells/µL (LQ 339 cells/µL–UQ 469 cells/µL). All patients had CD4 cell count >200 cells/µL, 14 patients had CD4 cell count >350 cells/µL. Fifteen patients demonstrated an HIV viral load below 50 copies/mL. The highest HIV viral load amounted to 385 000 copies/mL.

The comparison between patients with and without HIV/HCV co-infection is depicted in table 1. In the group of co-infected patients, a higher incidence of infection with HCV genotypes 3 and 4, and a lower incidence of infection with genotype 1, was observed compared to the group with HCV mono–infection.

**Table 1:** Selected demographic, laboratory and histo-pathological features of subjects.

	HIV/HCV co-infection		HCV mono-infection		P
	LQ-UQ	MEDIAN	LQ-UQ	MEDIAN	
Age years	29–34	32	21–48	29	>0.05
ALT U/L	44–83	54	39–83	54	>0.05
HCV viremia×10^6^ IU/mL	0.39–4.56	1.68	0.39–6.23	2.19	>0.05
	**Number of patients**	**fraction**	**Number of patients**	**fraction**	**P**
age≥40 years	2	9.52	20	28.99	>0.05
age<40 years	19	90.48	49	71.01	
Women	7	33.33	20	28.99	>0.05
Men	14	66.67	49	71.01	
HCV viremia >600 000 IU/mL	13	61.90	45	65.22	>0.05
HCV viremia ≤600 000 IU/mL	8	38.10	24	34.78	
IL28 B genotypes:					
CC	6	28.57	22	31.88	>0.05
TT	3	14.29	12	17.39	>0.05
CT	12	57.14	35	50.72	>0.05
ALT≥50 U/L	13	61.90	39	56.52	>0.05
ALT<50 U/L	8	38.10	30	43.48	
G≥2	3	14.29	20	28.99	>0.05
G<2	18	85.71	49	71.01	
S≥2	15	71.43	50	72.46	>0.05
S<2	6	28.57	19	27.54	
HCV genotype 1	6	0.29	63	0.91	= 0.00000
HCV genotype 3	6	0.29	6	0.09	= 0.018973
HCV genotype 4	9	0.42	0	0.00	= 0.00000

ALT - alanine aminotransferase, in liver biopsy: G- grade of inflammation and necrosis, S-stage of fibrosis.

### HMOX1

HMOX1 expression was analyzed in 90 liver biopsy samples. Eight specimens were rejected because of the insufficient RNA amount or quality (A260/A280<1.8).

**Figure 1:**
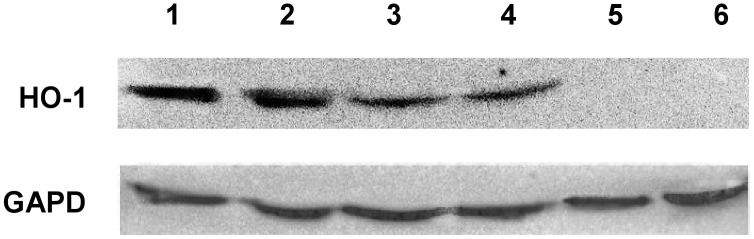
Presence of HO-1 protein levels for representative patients (Western Blot analysis).

A lower expression of HMOX1 was observed in patients co-infected with HIV/HCV. Expression of this gene was also lower in patients with lower HCV viral load (table 2). As regards the studied genotypes, a lower expression of HMOX1 was detected in patients infected with HCV genotype 4 in comparison with patients infected with HCV genotype 1. Results of the W-Blot confirm the described correlations (figure 1). While a strong band for HO-1 was observed in 4 out of 5 subjects with HCV viremia higher than 600 000 IU/ml, a weak band for HO-1 was observed in 3 out of 5 patients with HCV viremia below 600 000 IU/ml.

**Table 2:** HMOX1 expression depending on the studied parameters.

HMOX1MedianN = 82		P
Women	53.78	>0.05
Men	72.63	
Co-infection	27.74	= 0.0204
No co-infection	78.65	
Age≥40 years	61.35	= 0.05
Age<40 years	102.04	
HCV viremia >600 000 IU/mL	111.69	= 0.0006
HCV viremia ≤600 000 IU/mL	35.80	
*IL-28B* CC	81.10	>0.05
*IL-28B* not CC	65.97	
ALT≥50 U/L	62.16	>0.05
ALT<50 U/L	84.66	
G≥2	53.78	= 0.0495
G<2	106.98	
S≥2	72.55	>0.05
S<2	45.90	
HCV genotype 1	91.68	= 0.0274*
HCV genotype 3	26.36	
HCV genotype 4	17.50	

ALT - alanine aminotransferase, genotypes of IL28B: CC and not CC (CT and TT). In liverbiopsy: G- grade of inflammation and necrosis, S-stage of fibrosis.*Genotype 1 versus 3 p = 0.0501, genotype 1 versus 4 p = 0.0344, genotype 3 versus 4 p>0.05.

Figure 1 depicts the results of selected patients Only in some patients with co-infection no visible bands of HO-1 were seen (lane 5,6). Anti GAPDH antibody was used to confirm equal presence of cellular material in each sample.

### Subgroup of Patients with Co-infection

Higher expression of HO-1 was found in patients with CD4 count >350 cells/µl and HIV viral load <50 copies/ml (table 3). The presented results of the W-Blot confirmed this correlation on the protein level.

**Table 3:** HMOX1 depending on HIV viral load and CD4 count.

	HMOX1 MedianN = 21	P
HIV viremia<50 copies/mL	52.59	= 0.046
HIV viremia≥50 copies/mL	3.29	
Lymphocytes CD4>350 cells/µL	77.32	= 0.05
Lymphocytes CD4≤350 cells/µL	8.13	

All nine patients who had both CD4 count higher than 350 cells/ul and undetectable HIV viremia as well as all 6 patients with detectable HIV viremia were qualified for the W-Blot analysis.

### In the First Subgroup a Band for HO-1 was Observed in 8 out of 9 Patients

No band was observed in patients with CD4 count below 350 cells/ul and detectable HIV viremia.

**Figure 2:**
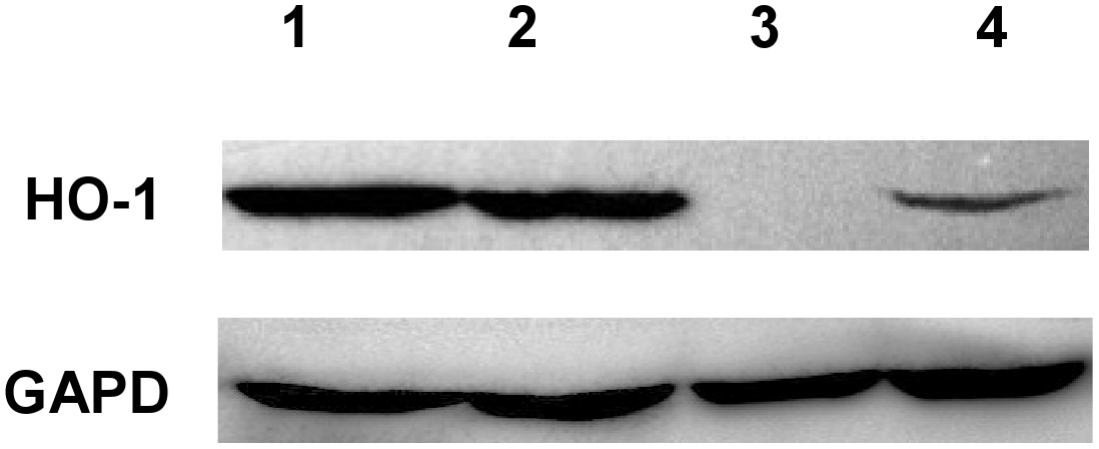
HO-1 protein levels in selected patients with HIV/HCV co-infection (Western Blot analysis).

Figure 2 depicts the results of selected patients with HIV/HCV co-infection, but different CD4 counts and HIV viral loads. Bach-1 has not been confirmed to have any inhibitory effect of on HO-1 expression.

Bach-1 expression was analyzed in first 60 liver biopsy samples. Two specimens were rejected because of the insufficient RNA amount or quality (A260/A280<1.8).

**Figure 3:**
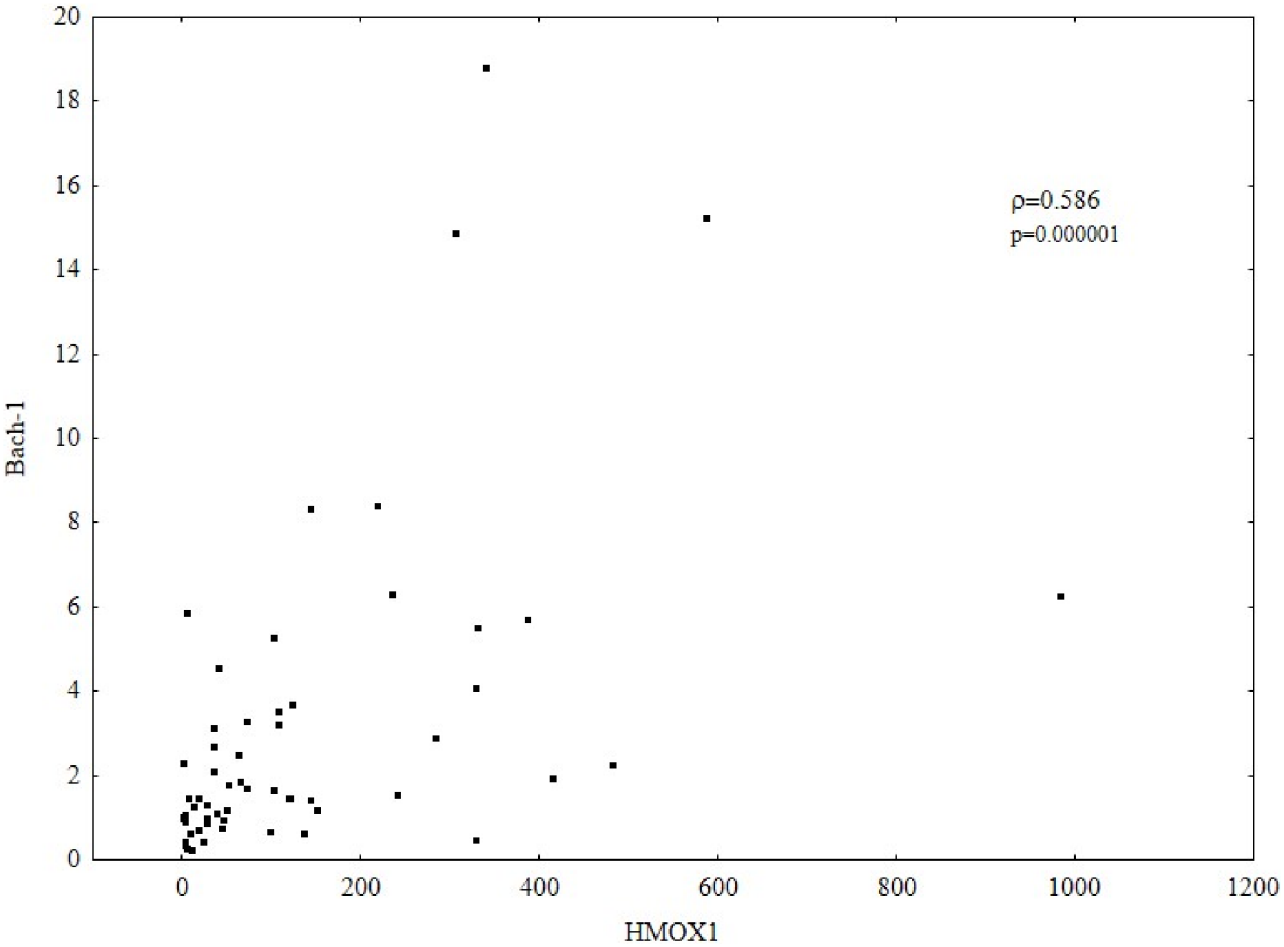
Correlation between the expression of HMOX1 and Bach-1. Gene expression was analyzed by relative quantitative (RQ) real-time PCR. mRNA levels were normalized to ACTB. Higher HMOX1 expression correlated with a higher expression of Bach-1 (Spearman’s rank correlation coefficient; ρ = 0.586, p = 0.000001).

**Figure 4:**
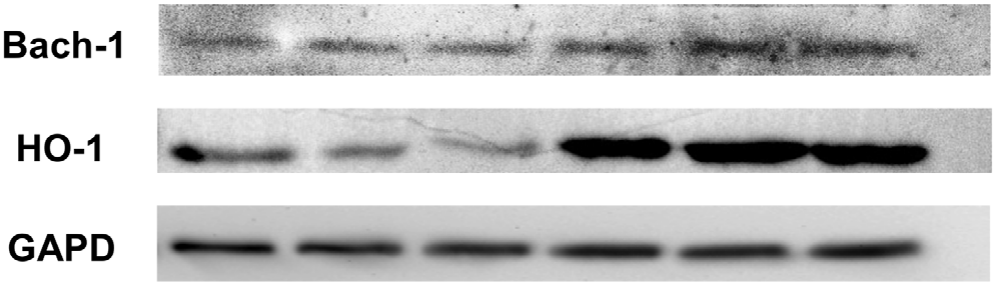
Positive correlation between HO-1 and Bach-1 protein level (Western Blot analysis).

The results of gene expression indicate a positive correlation between HO-1 and Bach-1. Higher HMOX1 expression correlated with a higher expression of Bach-1(Spearman’s ρ = 0.586, p = 0.000001) figure 3. Such a correlation was also confirmed by the W-Blot analysis, and selected results are presented in figure 4. Both in patients with mono- and coinfection, a weak band for Bach-1 was associated with a weak band for HO-1, and a strong band for Bach-1 was observed in the presence of a strong band for HO-1. Similarly, in coinfected patients in whom no band for HO-1 was observed, no band for Bach-1 was found.

### miR-122

**Figure 5:**
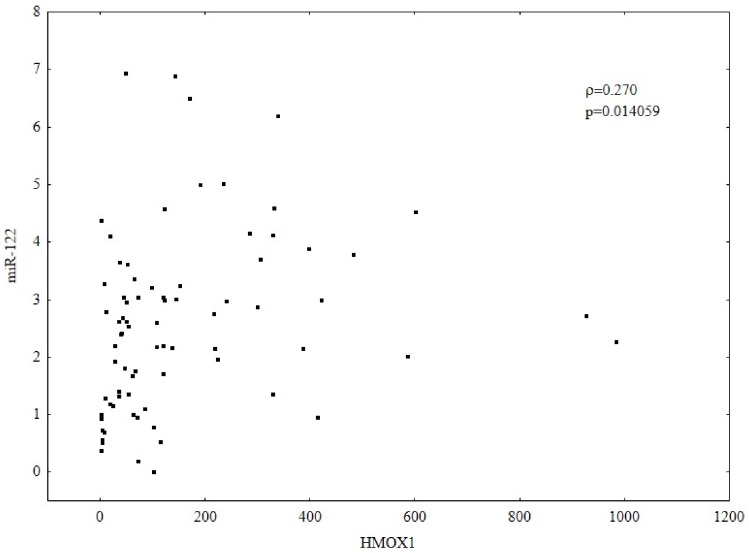
Correlation between the expression of HMOX1 and miR-122. Gene expression was analyzed by relative quantitative (RQ) real-time PCR. mRNA levels were normalized to ACTB for HMOX1 and to RNU24 for miR-122. Higher HMOX1 expression correlated with higher expression of miR-122. (Spearman’s rank correlation coefficient; ρ = 0.270, p = 0.014059).

**Figure 6:**
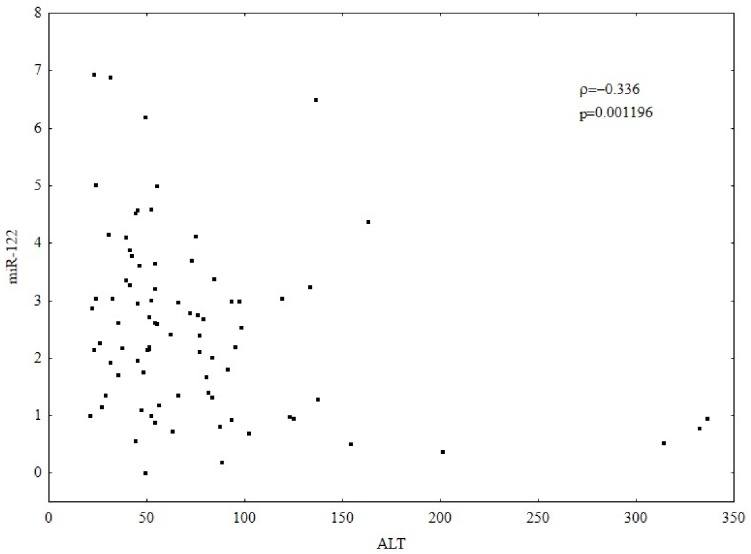
Correlation between ALT activity (U/L) and miR-122 expression. Gene expression was analyzed by relative quantitative (RQ) real-time PCR. mRNA levels were normalized to RNU24 for miR-122. Higher ALT activity correlated with a lower expression of miR-122 (Spearman’s rank correlation coefficient; ρ = −0.336, p = 0.001196).

The expression was found to be higher in patients with ALT <50 U/L than those with higher ALT activity (table 4). Higher HMOX1 expression correlated with a higher expression of miR-122 (ρ = −0.336, p = 0.001196) figure 5. Higher ALT activity was associated with a lower expression of miR-122 (ρ = −0.336, p = 0.001196) figure 6. No correlation between HCV viral load and miR-122 expression was observed.

**Table 4:** MiR-122 expression depending on the studied parameters.

MiR-122MedianN = 90		P
Women	3.22	>0.05
Men	2.20	
Co-infection	2.19	>0.05
No co-infection	2.73	
Age≥40 years	1.97	>0.05
Age<40 years	2.80	
Viral load>600 000 IU/mL	2.61	>0.05
Viral load ≤600 000 IU/mL	2.66	
*IL-28B* CC	2.84	>0.05
*IL-28B* not CC	2.52	
ALT≥50 U/L	2.41	= 0.0453
ALT<50 U/L	3.00	
G≥2	2.41	>0.05
G<2	2.69	
S≥2	2.76	>0.05
S<2	2.20	
HCV genotype 1	2.63	>0.05
HCVgenotype 3	2.71	
HCV genotype 4	2.19	

ALT - alanine aminotransferase, genotypes of IL28B: CC and not CC (CT and TT). In liver biopsy: G- grade of inflammation and necrosis, S-stage of fibrosis.

## Discussion

The HCV core protein is considered to cause increased production of ROS by altering mitochondrial function [19]. Controlled ROS production might be beneficial because it supports the elimination of HCV [7]. On the other hand, excessive production of ROS can result in hepatocyte injury. Therefore it is crucial to maintain a balance between pro-and antioxidative processes. HO-1 is one of the principal antioxidative enzymes. It is a cytoprotective enzyme which catalyzes the degradation of heme to bilirubin, carbon monoxide and iron and is induced by its substrate, heme, and by other stress-induced molecules [13], [20], [21].

Some authors suggested that different HCV proteins induce different antioxidant defense responses in hepatocytes. Abdalla et al [22], [23] showed that the expression of HO-1 was reduced in cell lines that stably expressed HCV core protein but increased in cell lines over-expressing HCV-NS proteins. On the contrary, Ghazini [16] demonstrates that HO-1 is up-regulated in hepatoma cells expressing not only HCV core protein but also additional structural and nonstructural proteins.

HO-1 has been shown to interfere with HCV replication but this connection is not yet clear. Several studies indicate that HO-1 induction or overexpression in replicons inhibit HCV replication [14], [15], while others have specified that it might be the products of the reaction catalyzed by HO-1 that are responsible for this process. It has been demonstrated that both iron and biliverdin display antiviral activity [24]–[27]. However, on the contrary, Kakizaki et al [28] note that iron enhances hepatitis C virus replication in cultured human hepatocytes. The present study demonstrates that HMOX1 expression positively correlated with HCV viremia in patients with CHC, and that expression was higher in patients with HCV viremia values greater than 600 000 IU/mL than in those with lower levels.

The mechanism of HMOX1 up-regulation in HCV infection is not precisely known, although it may be the result of a protective antioxidant response to excessive ROS production. One of the models explaining the increased expression of HMOX1 in patients with CHC was presented by Ghazani et al [16], who postulate that during HCV infection, the expression of Bach-1 gene is decreased, which causes de-repression of HMOX1. Bach-1 downregulation might be connected with oxidative stress, and HMOX1 induction could be a part of the response that hepatocytes use to protect themselves against oxidative stress.

However, the results of the present study do not indicate that a higher expression of Bach-1 is associated with a lower expression of HMOX1. On the contrary, Bach-1 positively correlated with HO-1. The W-Blot analysis provided similar results: patients in whom no band for HO-1 was observed also had a weak or absent band for Bach-1 and conversely, in those who had a strong band for HO-1, a band for Bach-1 was also found. Hence Bach-1 does not seem to play a role in the repression of HMOX1 in patients infected with HCV.

MiRNAs are post-transcriptional regulators which bind to complementary sequences on target messenger RNA transcripts. Several hundred miRNAs have already been distinguished and each of them is considered to regulate hundreds of target mRNAs. MiR-122, the most abundant miRNA in the liver, is known to positively regulate replication of the HCV in cell culture [29], [30]. However, no correlation between the expression of miR-122 and HCV viremia in patients with CHC was identified in the present study. Similarly, Sarasin-Filipowicz [17] report no positive correlation between miR-122 expression and intrahepatic and serum viral load in patients with CHC.

Some papers suggest that the antiviral effects of IFN in treatment of patients with CHC may be connected with the influence of IFN on miR-122. Pedersen [31] confirms that IFN-beta stimulation of Huh7 cells leads to a significant reduction in the expression of the liver-specific miR-122. However, Sarasin-Filipowicz [17] reports that the level of miR-122 in liver biopsies did not change significantly between before starting pegylated IFN-α treatment and 4 h afterwards. Interestingly, a study by Sarasin-Filipowicz [17] suggests that the role of miR-122 in controlling the HCV replication might be less important *in vivo* than *in vitro*.

It is worthy of note that miRNAs can also be involved in the regulation of HO-1. In our study, a positive correlation between miR-122 and HMOX1 was observed. Higher HMOX1 correlated with higher miR-122 expression. Additionally, it can be seen that a higher expression of HMOX1 implies a higher HCV viremia. Our results may suggest that in patients with CHC, miR-122 can regulate HCV replication partly through modulation of HMOX1 expression. Another possible explanation of the correlation between miR-122 and HMOX1 is that miR-122 indirectly increases oxidative stress and consequently leads to a protective increase of HMOX1 expression by affecting HCV replication. However, a fuller investigation of these correlations should be the subject of later studies.

The expression of HMOX1 was found to be higher in patients with lower gradings in liver biopsy specimens (G<2), which may suggest that the enzyme plays a protective, anti-inflammatory role. Conversely, HMOX1 expression was lowest in patients with HIV/HCV co-infection, especially those with lower CD4 lymphocyte counts and higher HIV viral loads, which is disadvantageous.

Lower expression of HMOX1 in patients with HIV/HCV co-infection can indicate a weaker antioxidative response to oxidative stress in this group of patients. Some authors suggest that HO-1 induction may counteract the effects of lipopolysaccharides (LPS) and proinflammatory cytokines in HIV infection [32].

## Conclusion

Reduced expression of HMOX1 in patients with HIV/HCV co-infection may indicate a worse prognosis in this group. Our results do not support the importance of Bach-1 in repression of HMOX1 in patients infected with HCV.
